# Evaluation of the Anticancer Properties of Geranyl Isovalerate, an Active Ingredient of *Argyreia nervosa* Extract in Colorectal Cancer Cells

**DOI:** 10.3389/fphar.2021.698375

**Published:** 2021-09-20

**Authors:** Fayyaz Rasool, Deepu Sharma, P. Shanmukha Anand, SKJ Magani, Srinivasan Tantravahi

**Affiliations:** ^1^ Department of Life Sciences, School of Natural Sciences, Shiv Nadar University, Greater Noida, India; ^2^ Department of Biotechnology, GITAM Institute of Science, Gandhi Institute of Technology and Management (GITAM) Deemed to be University, Visakhapatnam, India; ^3^ Department of Botany, Indira Gandhi National Tribal University, Amarkantak, India

**Keywords:** geranyl isovalerate, anticancer activity, HCT116 cell line, apoptosis, membrane potential

## Abstract

Chemotherapy is a general treatment procedure for cancer. The diversity in cancer incidence and the failure of therapy due to chemoresistance lead to increased cancer-related deaths. Therefore, new drugs with fewer secondary complications targeting diverse pathways are the need of the hour. Geranyl isovalerate (GIV), one of the active ingredients of ethyl acetate fraction of *Argyreia nervosa* is routinely used as a food flavoring agent. In this study, we found that GIV also exhibits anticancer activity when tested against the HCT116 cell line. It influenced the viability of the cells in a dose- and time-dependent manner. We examined whether GIV could induce oxidative stress and affect the mitochondrial membrane potential, thereby leading to apoptosis induction. Moreover, GIV could suppress the expression of antiapoptotic genes, such as BCl2 and PARP, and induce the expression of proapoptotic genes, such as Caspase 3 and 9. This is the first study demonstrating the anticancer activity of GIV and providing evidence for its mechanism of action. In conclusion, this study proposes GIV as a potential lead or supplementary molecule in treating and preventing colorectal cancer (CRC). Based on our findings, we conclude that GIV may be a viable lead or supplementary molecule for treating and preventing CRC.

## Introduction

Cancer is the second major cause of death worldwide after cardiovascular disease. The number of deaths due to cancer has been estimated to increase to 14.6 million by 2035. Colorectal cancer (CRC) ranks as the second most diagnosed cancer in women and the third most diagnosed cancer in men. However, deaths due to CRC in women are approximately 25% lower than in men (Dekker et al., 2019). CRC is the fourth most fatal malignancy worldwide, accounting for almost 0.9 Million deaths per year. Risk factors that can increase the incidence of CRC include inflammatory bowel disease, obesity, large intake of processed foods with high calories, and a lack of exercise leading to type II diabetes (Center et al., 2009). Chemotherapy is a general treatment procedure for any cancer, including CRC. The molecular diversity in cancer incidence and progression along with increased chemoresistance demands novel therapeutics for cancer. Phytochemicals have been a prominent source of anticancer agents that are used in clinics today. Traditional medicinal herbs are regarded as one of the major source of pharmacologically active compounds (Fridlender et al., 2015). Some of the plant-derived therapeutic molecules used in present-day clinics include vinblastine, vincristine, topotecan, irinotecan, etoposide, and paclitaxel (Cragg and Newman, 2005). The importance of biologically active phytochemicals and natural compounds drew the attention of scientists, and it has been reported that in 2014 alone 3,26,000 new natural compounds had been discovered (Banerjee et al., 2015). Out of these 3,26,000 compounds, only around 5% have been found to be effective. Therefore, investigation for new drugs targeting diverse pathways plays an important role in the success of cancer chemotherapy.

*Argyreia nervosa* (Burm.f.) Bojer, a popular herbal high (Paulke et al., 2014), is known to exhibit multiple medicinal properties, such as antimicrobial, immunomodulatory, anti-inflammatory (Bansal and Bharati, 2014), antihyperglycemic (Hema Latha et al., 2008), antidiarrheal, antitumor (Sharma et al., 2015), analgesic, and others (Lalan et al., 2015). Organic fractions exhibit most of the above activities. Ethyl acetate fraction in an earlier study by the authors showed immunostimulatory activity in Balb/c mice (data not shown). On evaluation of the ethyl acetate fraction by gas chromatography–mass spectrometry (GC-MS) studies, geranyl isovalerate (GIV) was identified as one of its major components (Supplementary Data). GIV was the least investigated ethnopharmacological compound of the extract, with only one commercial application as a food additive. In all the scientific reports till date, GIV has been mostly mentioned as a constituent of essential oil without any specific activity assigned to it (Rajeswara Rao et al., 2000; Couladis et al., 2001; Giannoulis et al., 2020). This is the first study reporting a bioactive effect of the compound. GIV is used as a food flavoring agent (WHO report, 1997). This chemical, when consumed, might come in direct contact with the cells of the gastrointestinal epithelium. With this basic idea, we chose HCT116, a colon cancer cell line, for the evaluation of the anticancer properties of GIV in this study. Monoterpenes, geraniol, and geranyl acetate, which are structurally similar to GIV, have been reported to exhibit anticancer activity against Colo-205, a colon cancer cell line (Zhang et al., 2018). This study is designed to evaluate the anticancer properties of GIV in colon cancer cell lines.

## Materials and Methods

### Isolation of Geranyl Isovalerate

Shade dried *A. nervosa* leaf powder was extracted using water, ethyl acetate, and ethanol. The extracts were studied for immunomodulatory activity. The promising ethyl acetate extract was further analyzed by GC-MS for its components, where GIV was reported to be one of its major constituents. GIV used for this study was procured from Sigma-Aldrich (109-20-6), and various concentrations ranging from 500 µm to 5 mM were used to understand its antiproliferative activity and mechanism of action.

#### Cell Culture

HCT116 and HT29 epithelial adherent colon cancer cell lines were used in this study. These were grown in Dulbecco's Modified Eagle’s Medium (Gibco, Thermo Fisher Scientific, REF 11995-065) and 10% fetal bovine serum, 2 mM L-glutamine (Gibco, REF 10270-106), and 1% penicillin/streptomycin (HIMEDIA, REF A001-100ML) was used as a supplement. Cells were incubated at 37°C and 5% CO_2_ in a humidified incubator.

### Cytotoxicity Evaluation Using MTT Assay

Cytotoxicity of GIV was checked against the HCT116 and HT29 cells using the MTT (3-(4,5-dimethylthiazol-2-yl)-2,5-diphenyltetrazolium bromide) assay. Cells were harvested and seeded in a 96-well culture plate with a density of 1 × 10^4^ cells per well and incubated for 24 h. The cells were treated with different concentrations of GIV ranging from 0.5 to 8 mM for 48 h. After 48 h of treatment, cells were incubated with 50 µg of MTT (5 mg/ml, HIMEDIA, REF-TC191-1G) for 2 h. The formazan crystals formed were solubilized with 100 µl of dimethyl sufoxide (Fisher Scientific Prod. No. 12435), and the absorbance was recorded at 595 nm (Senthilraja and Kathiresan, 2015). The percent viability of the cells was calculated with respect to the untreated control cells. The standard procedure was used to calculate the IC_50_ of the cells.

### Live/Dead Detection by Propidium Iodide Exclusion Assay

Additionally, to corroborate the MTT experiment results, 6 × 10^4^ cells/well of the HCT116 and HT29 cell lines were harvested and seeded in a six-well cell culture plate for 24 h. The cells were then treated with different concentrations of GIV from 0.5 to 5 mM. After the treatment of cells for the respective incubation period, the cells were stained using propidium iodide (PI, HIMEDIA, REF ML067) at a concentration of 10 µg/µl and incubated for 15–20 min. The cells were washed with phosphate-buffered saline (PBS) and then imaged at ×20 magnification using a Leica fluorescence microscope (Vyas et al., 2021).

### JC-1 Staining for Mitochondrial Membrane Potential Loss

To understand the mechanism of cell death, changes in mitochondrial membrane potential (MMP) were studied using JC-1 staining (Invitrogen, REF M34152) of GIV-treated cells. Cells treated with GIV for 24 and 48 h were stained with JC1 at a final concentration of 2 µM and incubated for 15–20 min. Cells were washed with PBS, and the image was taken by using a Leica fluorescence microscope at ×20 magnification. The stained cells were also used for flow cytometric analysis. Carbonyl cyanide m-chlorophenyl hydrazine (CCCP, Invitrogen, REF M34152) was used as a positive control to check the MMP loss during apoptosis (Sivandzade et al., 2019).

### Reactive Oxygen Species Generation and Geranyl Isovalerate

Cellular reactive oxygen species (ROS) generation was evaluated by dichlorofluorescein (DCFH-DA) dye (G-Biosciences Cat. #RC1066). Cells treated with GIV for 48 h were stained with dichlorofluorescein dye to a final concentration of 1 µM and incubated for 20 min. The cells were then washed with PBS and imaged at ×20 magnification using Leica fluorescence microscope (Wu and Yotnda, 2011).

### Gene Expression Profiling of Apoptotic Genes by qRT-PCR Analyses

The HCT116 cells were seeded in a T25 flask and treated with GIV. RNA was isolated from the cells using total RNA isolation kit (Promega, REF Z6111). RNA was quantified using Nanodrop, and 1 µg of the total RNA was used to synthesize cDNA using the cDNA synthesis kit (Promega, REF A3800). The cDNA obtained was used as a template to check the expression of apoptosis-associated genes, such as Caspase3, Caspase9, Bcl2, and Parp (primers were purchased from Imperialls, ILS) using SYBR Green Master Mix (Promega, REF A6001). 18s rRNA was used as an internal control. The Ct values attained through q-PCR were used to analyze the expression of the genes (Jozefczuk and Adjaye, 2011).

### Western Blot Analysis of Apoptosis-Associated Proteins

#### Western Blot Analysis

To check the expression of apoptosis-associated proteins, HCT116 cells were seeded in a T25 flask and treated with IC_50_ concentration and a concentration lower than IC_50_ of GIV for 24 h. After incubation, cells were harvested, and total protein was isolated using mammalian protein lysis buffer. The concentration of the protein was quantified using the Bradford assay. About 20 µg of the total protein was loaded on SDS gel. The resolved proteins were transferred on to a PVDF membrane, and the membrane was blocked using 5% non-fat skimmed milk and then probed with the following antibodies: beta-actin antibody (1:5,000) (ImmunoTag, Cat No. ITT07018, S. Rabbit), cleaved Caspase-3 (1:1,000) (ImmunoTag, Cat No. ITC0004, S. Rabbit), cleaved Caspase-9 (1:1,000) (ImmunoTag, Cat No. ITC0013, S. Rabbit), anti-Bcl2 (1:1,000) (ImmunoTag, Cat No. ITM3041, S. Mouse), cleaved PARP (1:1,000) (ImmunoTag, Cat No. ITT07023, S. Rabbit), secondary Goat anti-Rabbit IgG/HRP (1:5,000) (ImmunoTag), Goat anti-Mouse (1:5,000) (Thermo Fisher, Product No. 31431) (Gwozdz and Dorey, 2017; Taylor et al., 2013).

### Statistical Analyses

All the data are the representations of mean ± SD of at least three different experiments. Student' *t*-test was performed to derive *p* values, and *p* < 0.05 was considered as statistically significant.

## Results and Discussion

### Cytotoxicity Study of Geranyl Isovalerate

**FIGURE 1:**
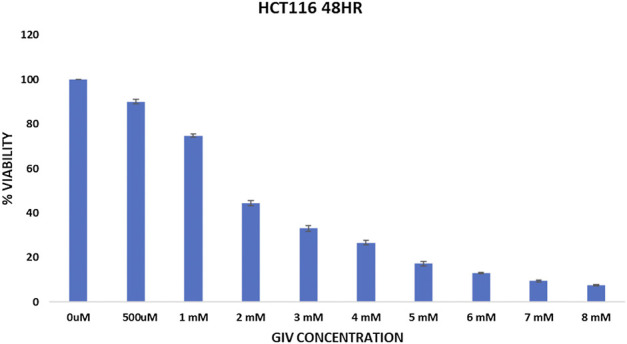
Cytotoxicity of GIV evaluated using MTT assay. GIV shows IC_50_ around 1.6–1.8 mM concentration. The x-axis denotes GIV concentration and y-axis represents percentage viability. Data are represented as mean ± SD values of three independent experiments for 48 h of incubation, and *p* value < 0.05 is considered significant.

Cytotoxicity of GIV was evaluated against two different CRC cell lines (HCT116 and HT29) using the MTT assay (Figure 1 and Supplementary Figure S2), which is a calorimetric assay to calculate the metabolic activity of the cell (Stockert et al., 2018). NADPH-dependent oxidoreductase enzyme in a healthy cell has the capability to reduce the tetrazolium dye—MTT (3-(4,5-dimethylthiazol-2-yl)-2,5-diphenyltetrazolium bromide)—to the insoluble purple dye called formazan (Senthilraja and Kathiresan, 2015). The higher the amount of formazan formed, the more viable the cells become. The IC_50_ value of GIV was found to be around 1.8 mM for HCT116 cells and approximately 4 mM for HT29 cells.

### Live and Dead Cell Detection

**FIGURE 2:**
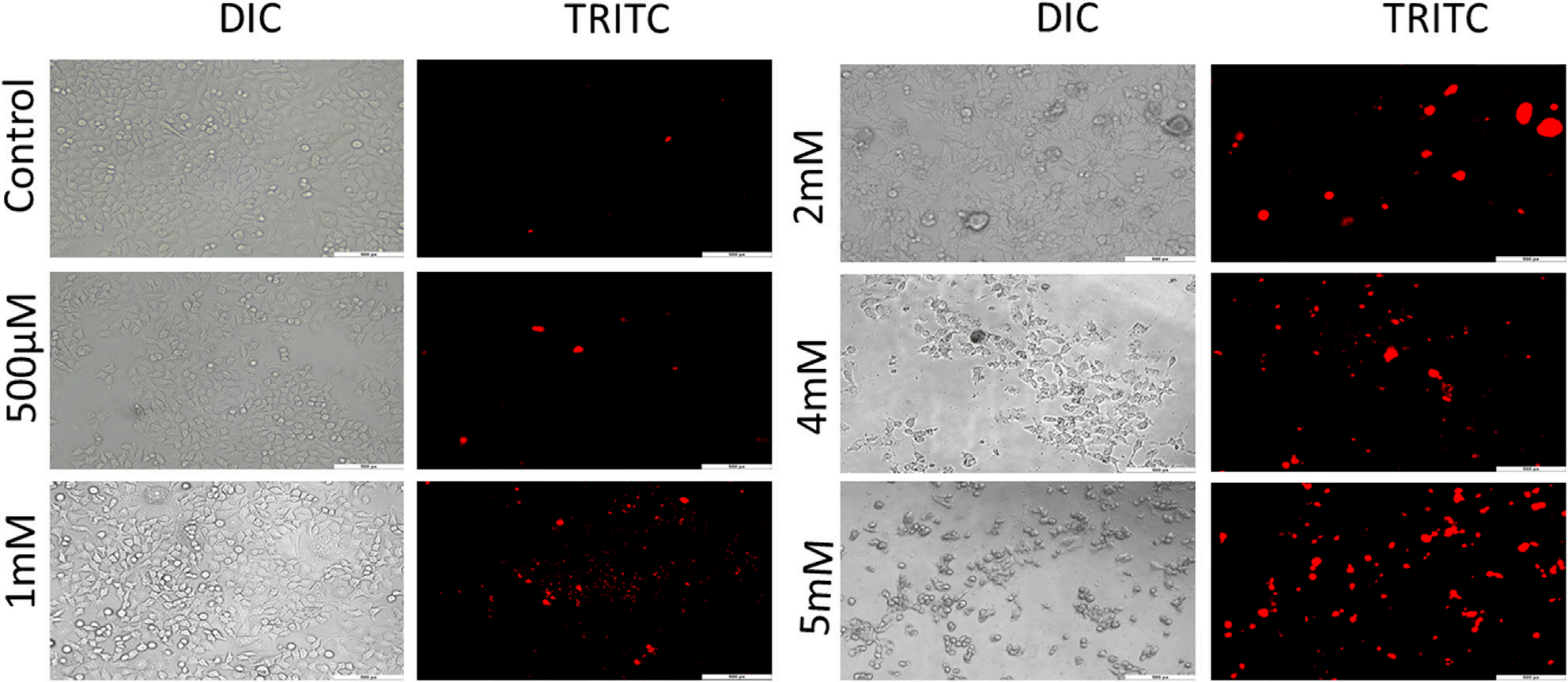
PI staining for live/dead exclusion. HCT116 cells treated with GIV show higher PI positivity, an indication of compromised cell membranes. The cells were imaged at ×20 magnification.

Further to confirm the cytotoxicity observed by the MTT assay, PI staining was performed for which images were captured under fluorescence microscope. PI is a membrane impermeant red fluorescent DNA intercalating dye. This dye can cross the membranes only when the membranes are compromised (Lukowiak et al., 2001). It is used to differentiate live/dead cells on the basis of membrane integrity. The loss of membrane integrity is one of the hallmark features of cell death. PI when bound to DNA excites at 493 nm and emits a fluorescent red light of 636 nm (Cummings and Schnellmann, 2004; Lukowiak et al., 2001). Cells treated with GIV exhibited an increase in red fluorescence in comparison to the untreated control cells. As shown in Figure 2 and Supplementary Figure S3, this increase in red fluorescence was observed to be concentration dependent. All these results of PI microscopy supported the results of cytotoxicity observed by using the MTT assay.

### JC-1 Staining

**FIGURE 3:**
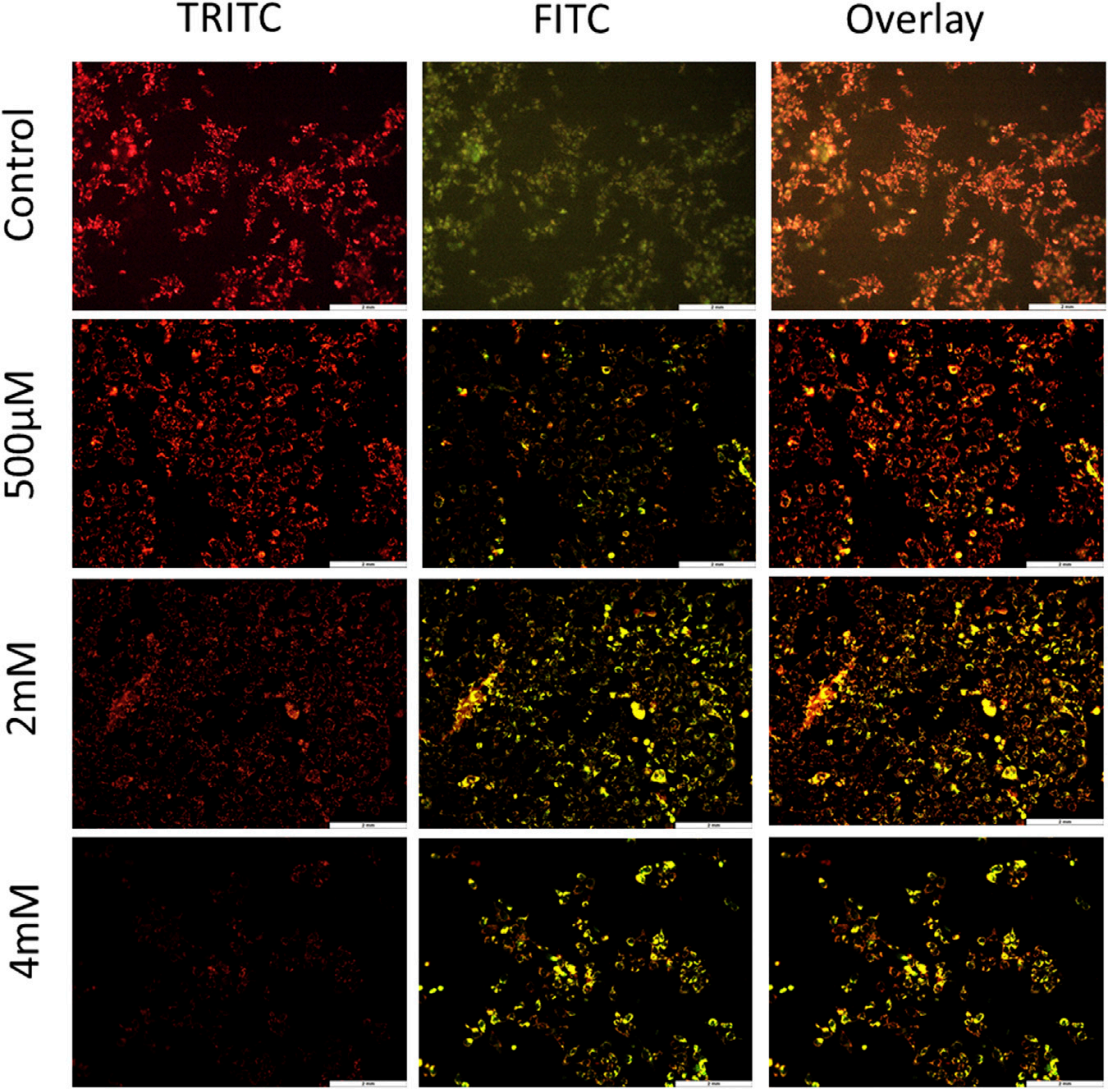
HCT116 cells treated with GIV and analyzed for MMP loss using JC-1 staining. Cells exhibit more green fluorescence with increasing GIV concentrations, indicating membrane potential loss. The cells were imaged at ×20 magnification.

To further confirm the process of cell death and contribution of the mitochondria in cell death, JC-1 staining was performed. The loss of MMP is one of the hallmark features of apoptosis. To evaluate the loss of MMP, JC-1—a lipophilic cationic dye—was used. In healthy cells, this dye gets accumulated in the negatively charged mitochondria, thus forming J-aggregates that emit red fluorescence. However, in unhealthy apoptotic cells, there is less negative charge inside the mitochondria due to membrane potential loss and therefore JC-1 dye enters the mitochondria but is unable to form stable J-aggregates. It exists in a monomeric form which emits green fluorescence (Sivandzade et al., 2019). For JC-1 stain imaging by fluorescence microscopy, TRITC and FITC channels were used, and both the images were overlayed to examine the concentration of J-aggregates verses J-monomers. As shown in Figure 3 and Supplementary Figure S4, cells treated with GIV clearly showed an increase in J-monomers, with increasing GIV concentration showing MMP loss. This indicates that GIV induces apoptosis through the mitochondria-mediated pathway. It has been reported that chemotherapeutic drugs are shown to induce MMP loss, thereby inducing apoptosis (Vyas et al., 2021).

**FIGURE 4:**
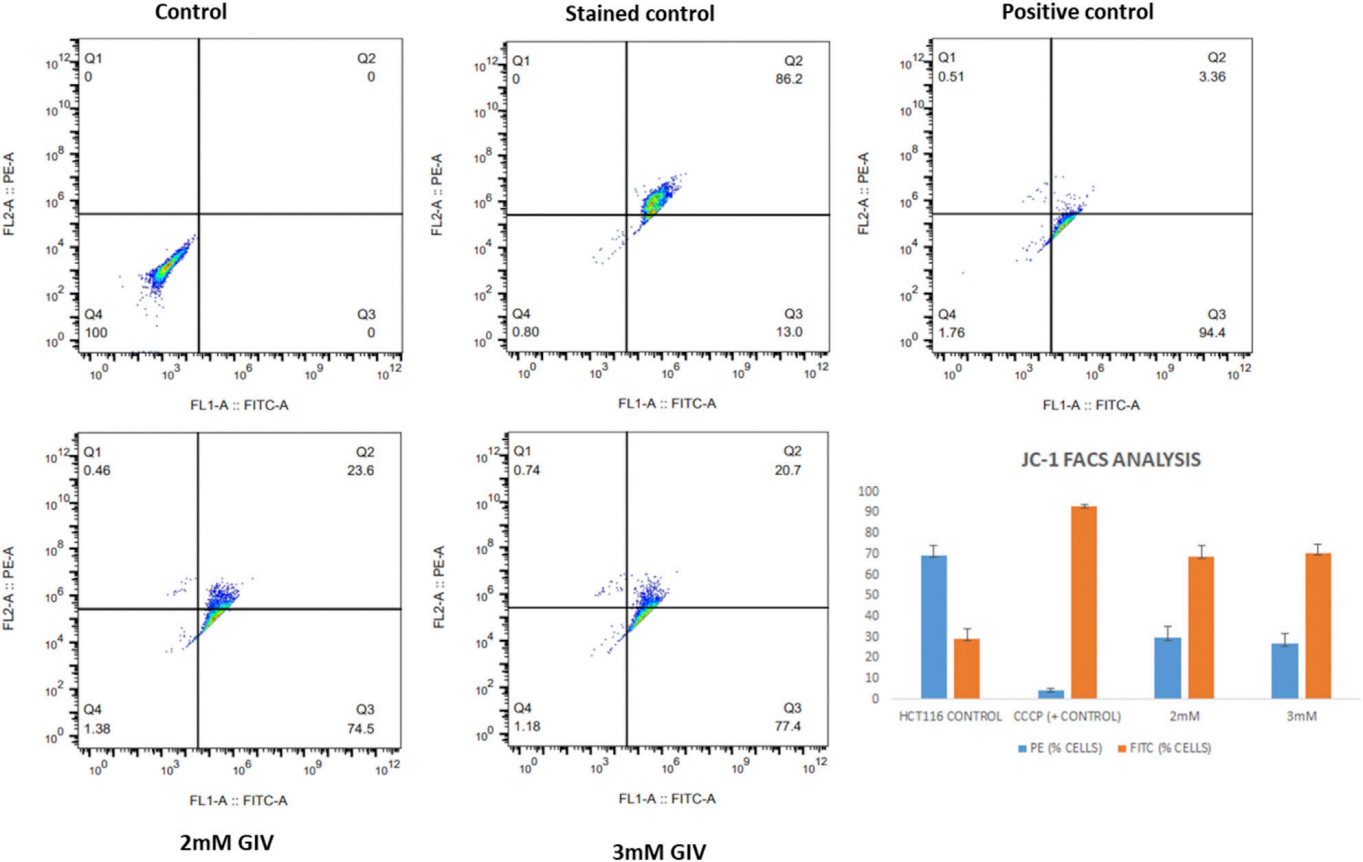
HCT116 cells treated with GIV and analyzed for loss of MMP by flow cytometry using JC-1 staining. PE and FITC-positive cells under different experimental conditions are represented in the bar diagram.

This depolarization of the mitochondria and the induction of apoptosis were also quantified by the flow cytometry experiment. As shown in Figure 4, there is an increase in cells in the FITC channel upon treatment with GIV, representing the loss of MMP. CCCP is used as a positive control that depolarizes the mitochondria in almost all the cells. The bar diagram in Figure 4 shows the extent of depolarization with different concentrations of GIV.

### Generation of Reactive Oxygen Species by Geranyl Isovalerate

**FIGURE 5:**
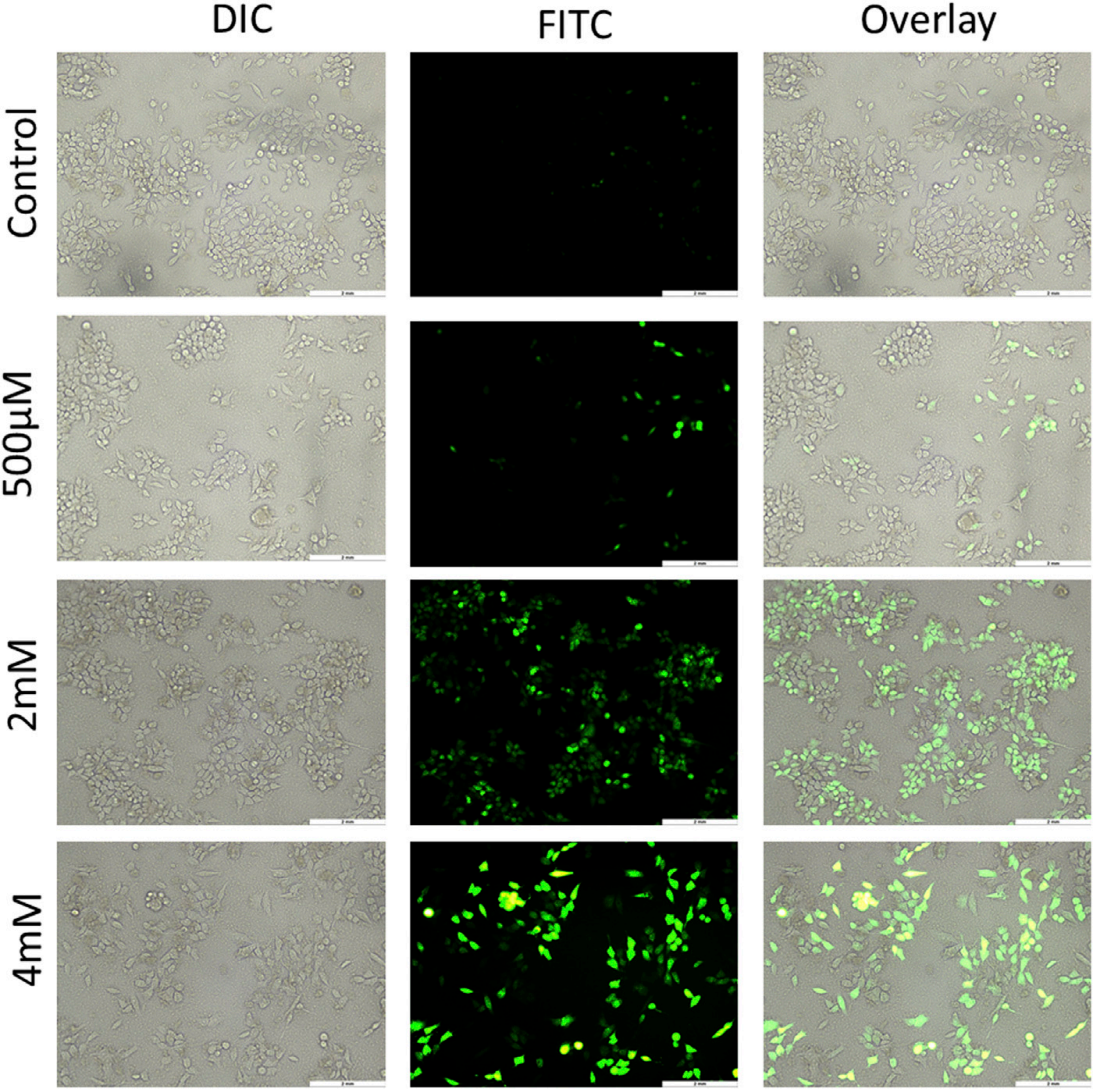
HCT116 cells treated with GIV, analyzed for ROS generation using DCFH-DA dye, and imaged at ×20 magnification. The cells show increased green fluorescence with increasing concentrations of GIV in comparison with the control cells, indicating ROS generation with GIV treatment.

ROS generally have a short life span and are highly reactive. At a moderate level, ROS are good for regulating normal physiological functions, and for maintenance of the redox balance and immune system. However, an excess of ROS damages nucleic acids, proteins, lipids, membranes, and organelles, leading to apoptosis-related cell death. ROS have an essential role in cell signaling and also regulate key pathways related to apoptosis, which are mediated by death receptors, the mitochondria, and the endoplasmic reticulum (Redza-Dutordoir and Averill-Bates, 2016). ROS generation has been reported to be a part of the activity of many chemotherapeutic drugs. There are various methods to measure free radical production in mammalian cells, but the use of 2ʹ-7ʹ-dichlorodihydrofluorescein diacetate (DCFH-DA) is the most common for this purpose. DCFH-DA is basically a cell-permeable, intracellular nonfluorescent precursor of DCF that is used as a probe to measure oxidative stress of cells. DCFH-DA gets hydrolyzed to DCFH that is impermeable and therefore remains inside the cell, which is finally oxidized to dichlorofluorescein that emits green fluorescence (Eruslanov and Kusmartsev, 2010). This DCF accumulation is measured by the increase in fluorescence at 530 nm by the FL-1 green channel of fluorescence microscopy. In the experiment of this study, HCT116 and HT29 cells treated with GIV showed an increase in green fluorescence in a concentration-dependent manner. As shown in Figure 5 and Supplementary Figure S5, the number of cells exhibiting green fluorescence increased with higher concentrations of GIV than did control cells. This indicates an increase in ROS generation upon treatment with GIV.

### Change in Expression of Apoptosis-Related Genes Induced by Geranyl Isovalerate

To check the apoptosis-related genes at the transcriptome level, gene expression profiling was performed in control HCT116 cells and 48-h drug-treated cells at concentrations of 1, 2, and 3 mM. BCl2, Caspase 3, Caspase 9, and PARP were used for expression profiling, and 18S RNA was used as a housekeeping gene. we continued our experiments with HCT116 cells alone as HT29 had an IC50 value at higher concentration IC_50_ value at higher concentration, so we continued our experiments with HCT116 cells alone.

Bcl2 protein family is a well characterized protein family which has an important role in regulating the intrinsic pathway of apoptosis. This family of proteins contain both proapoptotic and antiapoptotic members. Bcl2 is an antiapoptotic member of this protein family (Tsujimoto, 1998). Bcl2 protein has been shown to promote an increase in cancer cell population by preventing cell death and promote resistance to chemotherapy in cancer cells (Reed et al., 1996). It conserves the integrity of the mitochondrial membrane to inhibit apoptosis, as it contains a hydrophobic carboxyl-terminal domain that is linked to the outer mitochondrial membrane. Bcl2 interacts with proapoptotic BAX, thereby preventing oligomerization of BAX and BAK and their translocation to the mitochondrial membrane. The translocation of BAX–BAK oligomer to the mitochondrial membrane punches pores in the membrane, releasing various proapoptotic factors such as cytochrome C from the mitochondria. Thus increased Bcl-2 expression prevents the apoptosome complex formation, preventing the activation of downstream caspase cascade. Bcl-2 has been also shown to control the initiation of some initiator caspases such as caspse2, thereby preventing apoptosis (Guo et al., 2002).

Caspases are endopeptidases, which are widely divided into three groups, namely, initiator (caspase 2, 8, 9, and 10), executioner (caspase 3, 6, and 7), and inflammatory (caspase 1, 4, 5, 11, and 12). The initiator caspases activate the apoptotic signaling cascade; however, the executioner does the proteolysis of important proteins that is important for apoptosis (Role of caspases in apoptosis, 2021). Caspase 9 is an initiator caspase that activates downstream executioner caspases, thus initiating apoptosis. By forming an apoptosome, the seven APAF-1 subunits activate one caspase-9; the activated caspase-9 then cleaves-3,-6 and -7, Which in turn cleaves various cellular targets, resulting in (Kuida, 2000). Caspase-3 is an executioner caspase, which upon activation performs an effective function of/in apoptosis by degrading intracellular protein (Porter et al., 2021). Tetrandrine citrate shows antitumor activity in glioma cells by increasing the expression of caspase-3 and ROS generation, while decreasing Bcl2 expression (Sun et al., 2019). The data from earlier JC-1 staining and ROS generation point toward the involvement of the intrinsic pathway of apoptosis. As a result, the levels of caspase-9, the intrinsic pathway intiator caspase, and executionary caspase-3 were measured.

PARPs (poly ADP–ribose polymerases) are the enzymes that catalyse the transfer of ADP–ribose to target proteins. PARPs also have an important role in various biological processes inside a cell, such as chromatin structure modulation, replication, transcription, recombination, and DNA repair (Morales et al., 2014). PARP helps damaged cells to repair themselves, thereby playing an important role in cancer. Therefore, PARP inhibitors stop PARP from performing repair work, thereby making cancer cells die (Cancer Research UK, 2021). PARP cleavage is found to be associated with cell death. Therefore, the cleavage product enhances apoptosis and prevents cell survival (D’Amours et al., 2001).

**FIGURE 6:**
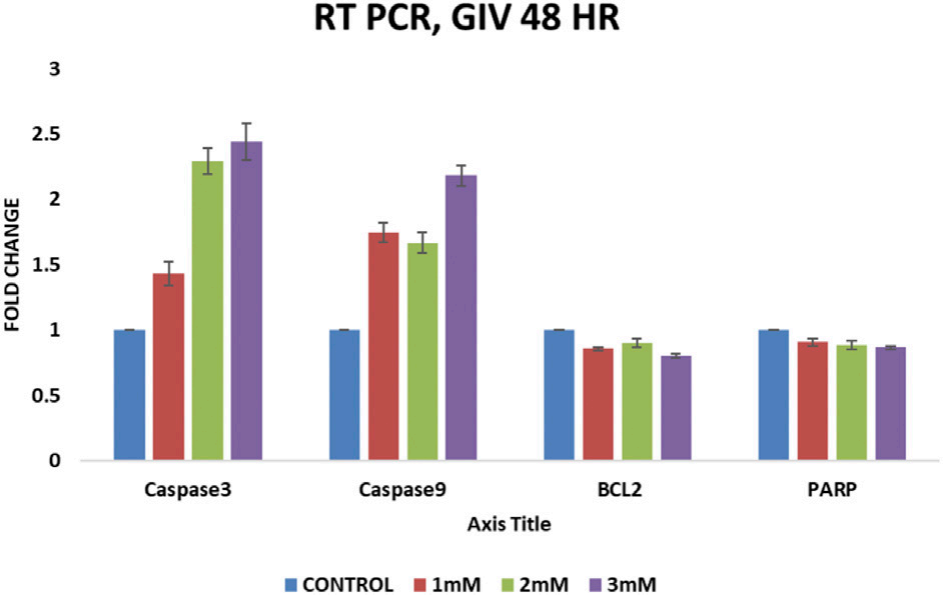
QPCR analysis of apoptosis-regulating genes Bcl2, Caspase-3, Caspase-9, and PARP. HCT116 cells treated with different concentrations of GIV show an increase in expression of proapoptotic caspase-3 and -9 and a decrease in expression of antiapoptotic Bcl-2 and PARP genes.

RT-QPCR experiments after GIV treatment show a significant increase in caspase-3 and caspase-9 levels in a concentration-dependent manner. However, a slight change in expression of Bcl-2 (antiapoptotic gene) and PARP (damaged cells repair gene) has been also observed, as shown in Figure 6. The increased expression of proapoptotic caspases and decreased expressions of antiapoptotic Bcl-2 and DNA repair–associated PARP further confirm apoptosis induction by GIV treatment.

### Change in Expression of Apoptosis-Related Proteins

**FIGURE 7:**
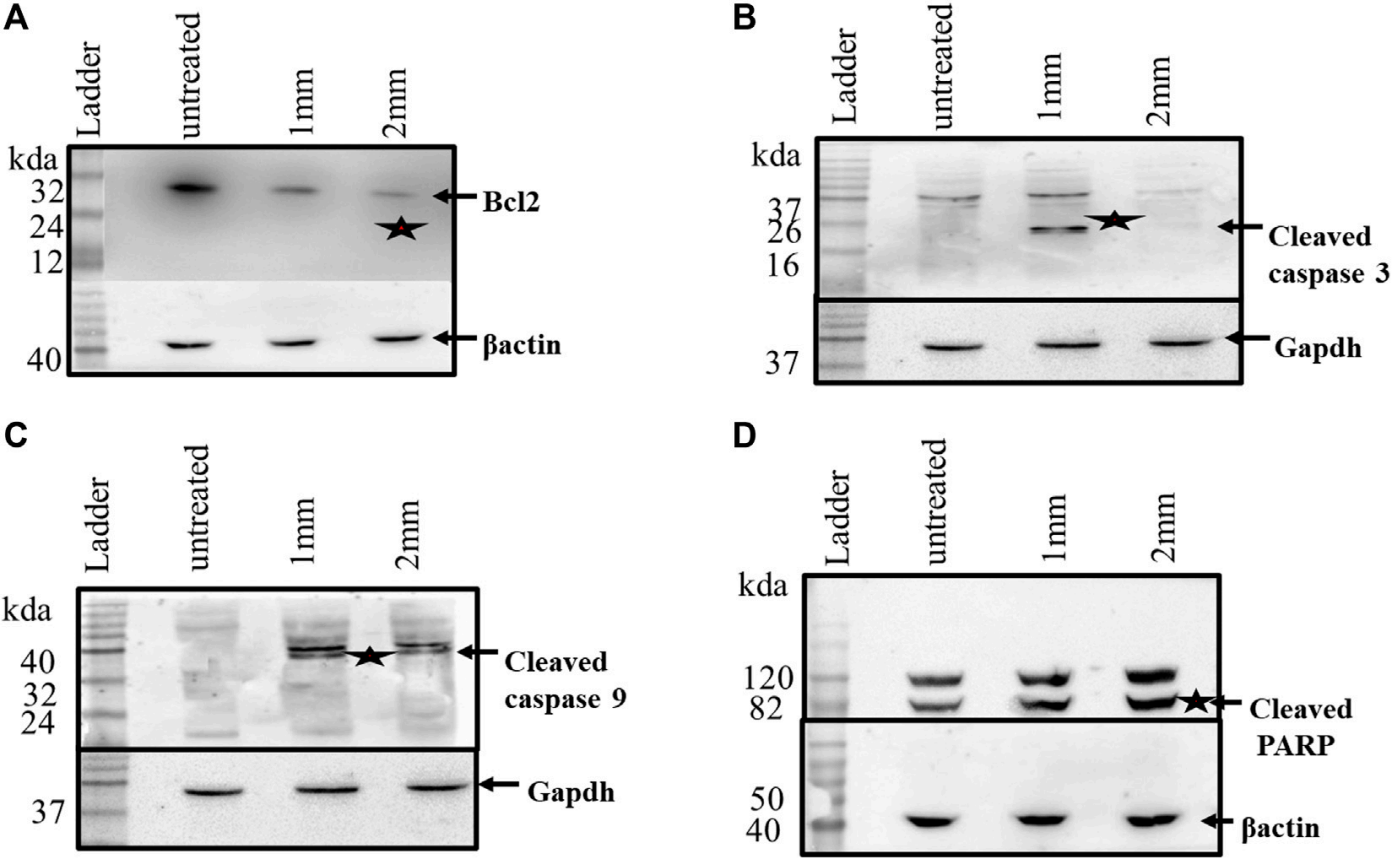
HCT116 cells treated with GIV and analyzed for expression of apoptosis-associated proteins. **(A)** Expression of Bcl2 in the control and GIV-treated cells. **(B)** Cleaved caspase-3 protein levels in the control and GIV-treated cells. **(C)** Cleaved caspase-9 protein levels in the control and GIV-treated cells. **(D)** Cleaved PARP levels in the control and GIV-treated cells. β-actin and GAPDH proteins have been used as the internal controls.

After investigating the expression of apoptosis-related genes at the transcriptional level, their expressions at the protein level were checked by western blot analyses. Caspases have been reported to exist in an inactive zymogen state in healthy cells, but upon induction of apoptosis, these inactive zymogens cleaved to form an active caspase. As shown in Figure 7, caspases3 and 9 show cleaved products, which is a feature of apoptosis. The western blot analyses further confirmed the decrease in Bcl-2 expression as observed in the Rt-PCR experiments. Cleavage of PARP is another important event during apoptosis, which was also reported after GIV treatment. The western blots further confirmed that GIV induces apoptosis by activation of caspases, decreasing the expression of Bcl-2. These results confirmed the involvement of an intrinsic pathway of apoptosis in cell death of GIV-treated cells. Furthermore, the morphological features of dying cells along with the expression changes of the Bcl-2, Parp, and Caspase genes support apoptosis rather than any other form of cell death.

## Conclusion

This study was performed to evaluate the anticancer properties of GIV, an active ingredient of *A. nervosa* extracts. To establish the anticancer properties of GIV, cytotoxicity studies, ROS generation, loss of MMP, and expression profiling of apoptosis-associated genes at the transcriptional and translational levels were evaluated. Based on the results, it can be concluded that GIV induces apoptosis through oxidative stress–mediated pathways in both the cell lines studied. The increased ROS generation, loss of MMP, decreased expression of antiapoptotic genes, and increased expression of proapoptotic genes supported oxidative stress–mediated cell death. GIV is already a WHO-approved food additive. Furthermore, *in vivo* experimental data are required to determine if this compound can be used as a food supplement along with other chemotherapeutic agents in order to see if this molecule can increase the efficacy of therapy. A detailed study is also required to understand if structural modifications of GIV could increase the efficacy of the compound and can be individually used as a therapeutic agent.

## Data Availability

The original contributions presented in the study are included in the article/Supplementary Material; further inquiries can be directed to the corresponding authors.
