# Supplementary figures and images for: Evaluation of the Anticancer Properties of Geranyl Isovalerate, an Active Ingredient of Argyreia nervosa Extract in Colorectal Cancer Cells
Source: Front Pharmacol. 2021 Sep 20;12:698375. doi: 10.3389/fphar.2021.698375 (PMC8489534; doi:10.3389/fphar.2021.698375)

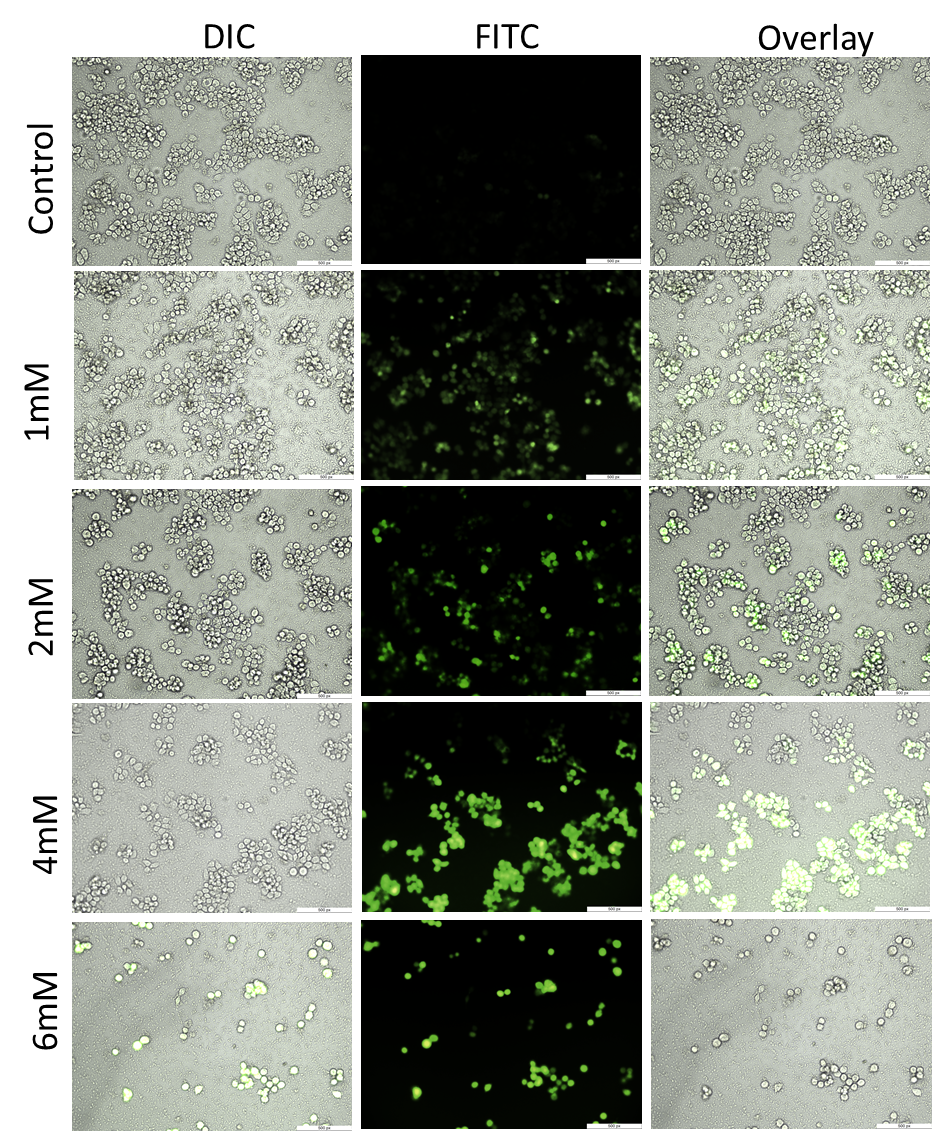

Supplement: Supplementary file 1 [file Image6.TIF]

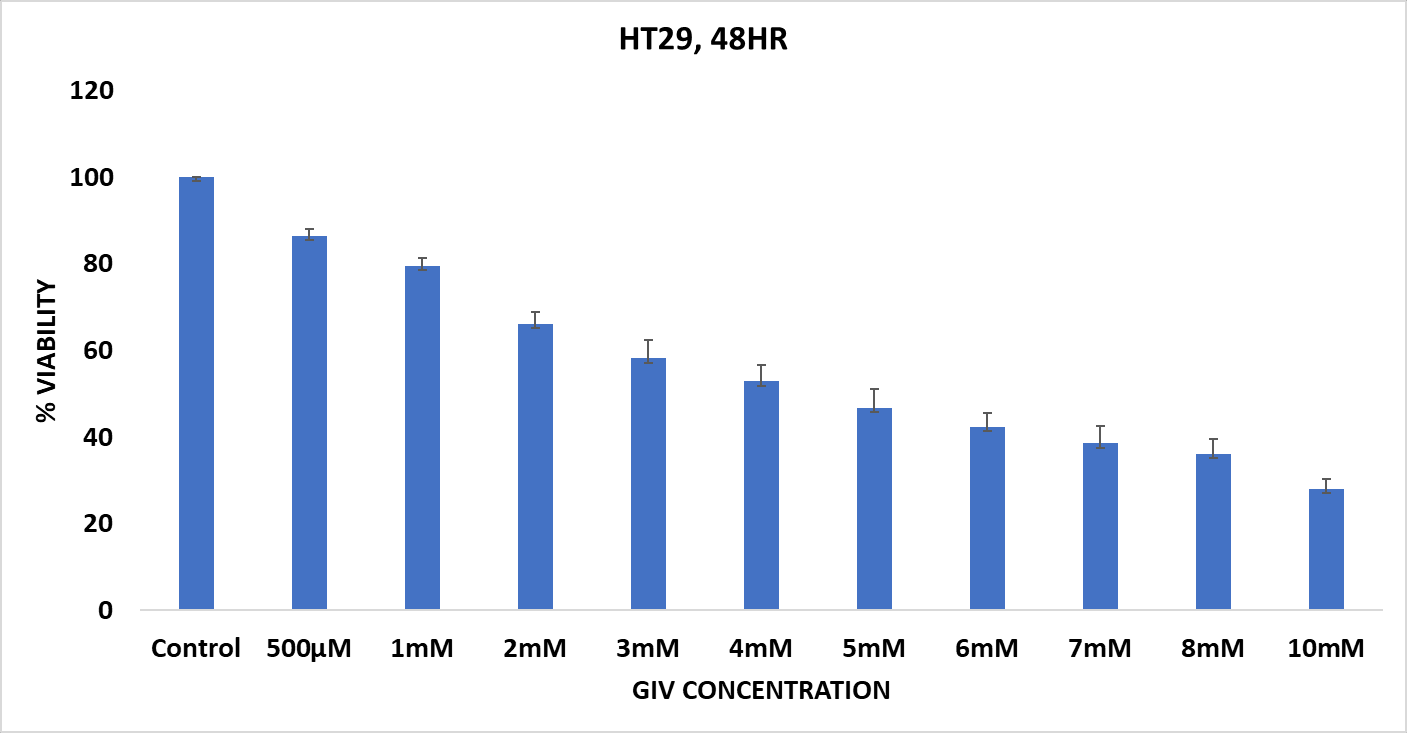

Supplement: Supplementary file 2 [file Image3.TIF]

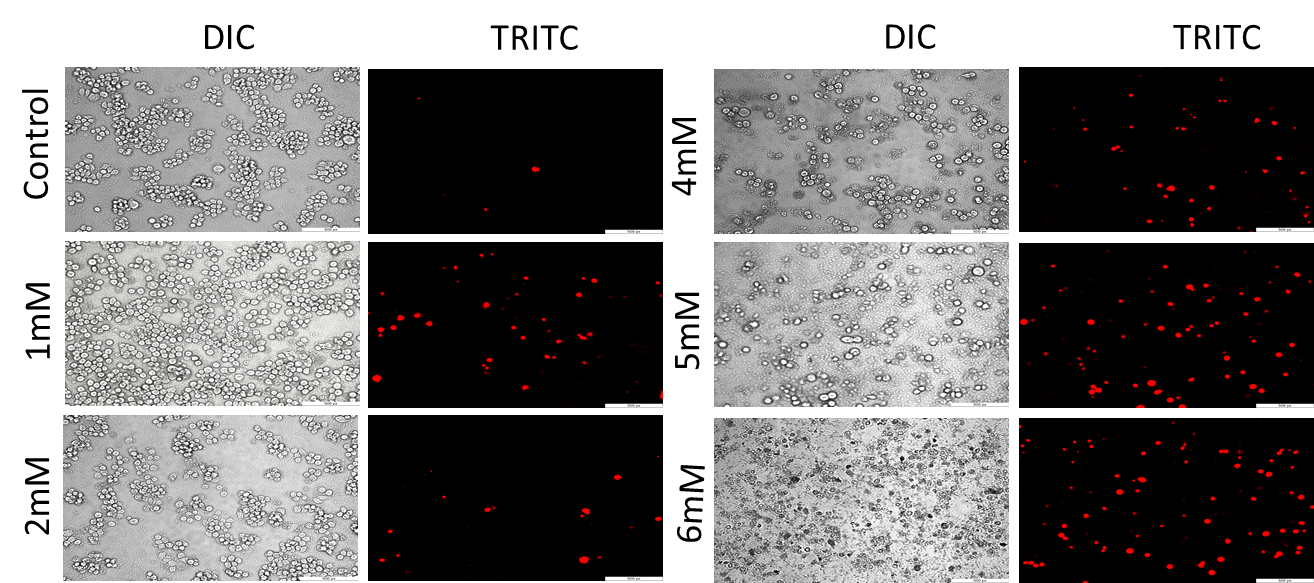

Supplement: Supplementary file 3 [file Image4.TIF]

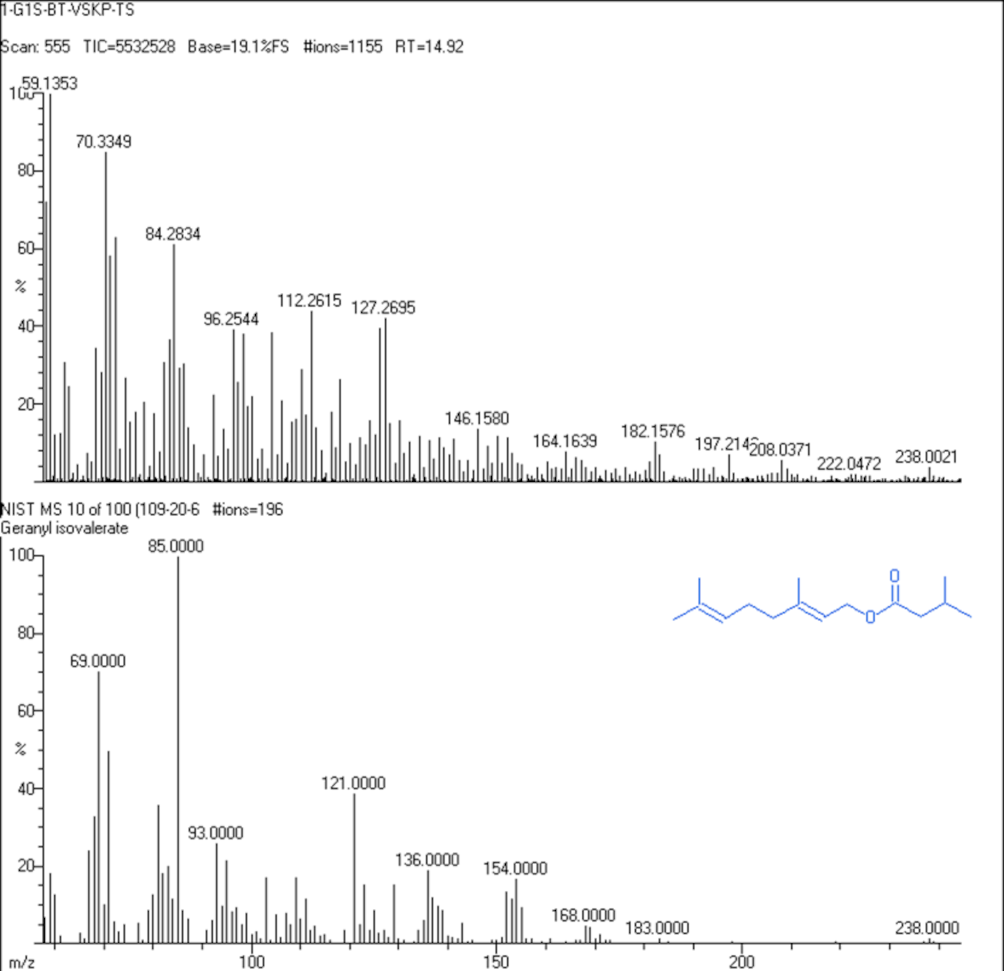

Supplement: Supplementary file 4 [file Image2.TIF]

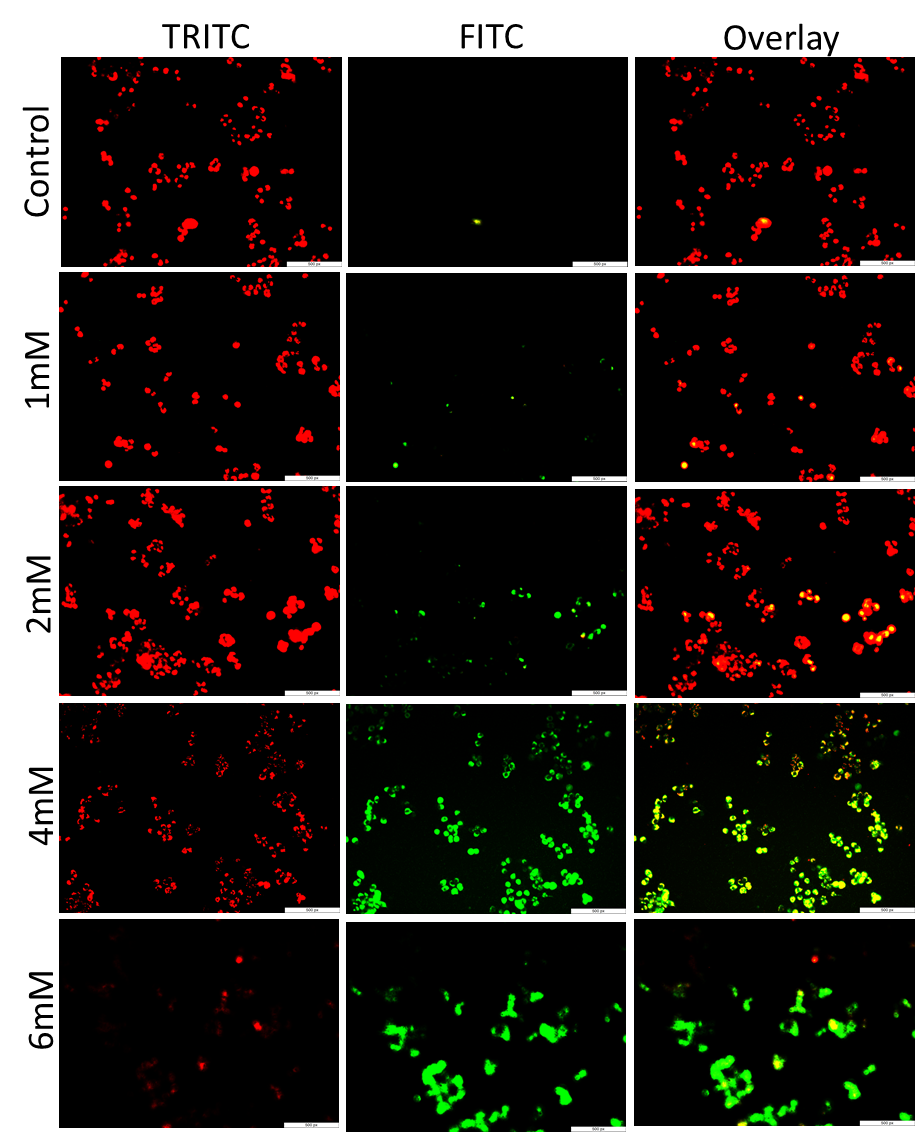

Supplement: Supplementary file 6 [file Image5.TIF]
